# Evaluating TOD performance through node-place model: evidence from bus stop areas in Windhoek, Namibia

**DOI:** 10.3389/fpubh.2025.1697702

**Published:** 2026-01-05

**Authors:** Nana Cui, Haochen Yang, Naholo Malakia Mwetuhafifa

**Affiliations:** School of Architecture and Design, Beijing Jiaotong University, Beijing, China

**Keywords:** bus-based TOD, equity, healthy city, Namibia, node-place mode, transit-oriented development (TOD)

## Abstract

**Background:**

Transit-oriented development (TOD) is now regarded as an important measure to improve sustainable urban mobility and land-use efficiency in rapidly growing cities. Despite the vast research on TOD implementation in the Asian and American context, little has been done regarding the application of TOD in African cities, especially those that are not equipped with metropolitan rails, such as Windhoek, Namibia.

**Methods:**

This study evaluates 135 bus stops in Windhoek based on a node-place (NP) model. Within a 400-meter walking catchment, three node indicators and seven place indicators are applied to define transport accessibility and land-use intensity in each instance each stop. The spatial analysis is carried out in GIS environment to combine NP indicators. The data are evaluated in three weighting schemes including Analytic Hierarchy Process (AHP), entropy weighting, and equal weighting to measure robustness and to determine the effects of weighting preferences on the NP index.

**Results:**

Most of the stops (80 out of 135) have place values that are higher than node values indicating common areas where the intensity of land-use is higher than the transport accessibility. There are clear north south differences in the values of Node-Place along the B1 road, with the city center being more node-place synergistic and the suburban regions being dependent in nature. These spatial differences are due to stage-specific planning paths. The sensitivity analysis demonstrates that AHP and equal weighting have high concordance, and entropy weighting has significant changes, with industrial land having a greater weight, and it would be recommended to have context-dependent calibration of indicators. The absence of “stress” areas presents a unique opportunity for proactive planning before congestion becomes entrenched. The discussion also indicates the possible health advantages of a better TOD such as more walking and transit, lower vehicle emissions, and easier access to health services, while acknowledging the low-income communities in the peripheral areas may face critical equity issues.

**Conclusion:**

The findings underscore the role of context-specific TOD planning in African developing cities, where typical stress conditions might not yet exist but systemic dependence issues require proactive intervention to prevent future imbalances.

## 1 Introduction

Cities around the world are experiencing unprecedented challenges, such as rapid urbanization, poverty, climate changes, pollution and the need for sustainable and inclusive development. These issues have an enormous impact on public health and well-being (1). Developing nations, in particular, are experiencing swift urban expansion, with the United Nations estimating that, by 2050, such countries as India, China and Nigeria will contribute 35 percent of the total urban population growth in the world (2). Namibia and its capital, Windhoek, in particular, have given an example of such tendencies, which creates a state of emergency in regards to studying the dynamics of urban areas and the strategy of planning.

Transit-Oriented Development (TOD) has been a well-known approach that has been proposed to resolve these problems by integrating land use and transportation planning (3). TOD is becoming increasingly appreciated as an opportunity to not only increase urban mobility and sustainability but also has a substantial co-benefit on the health of the population with the main benefits being in the promotion of active travel modes (walking and cycling), less reliance on personal vehicles (and the resulting air and noise pollution), and facilitating access to necessary services and amenities (4). Although TOD has been researched widely in the developed nations and parts of Asia and America, its application in the African cities has not been well researched. Cities of Africa have unique challenges in attaining inclusive, safe, resilient, and sustainable urban development (5, 6), thus, the need to plan TOD context-specifically.

**Figure 1:**
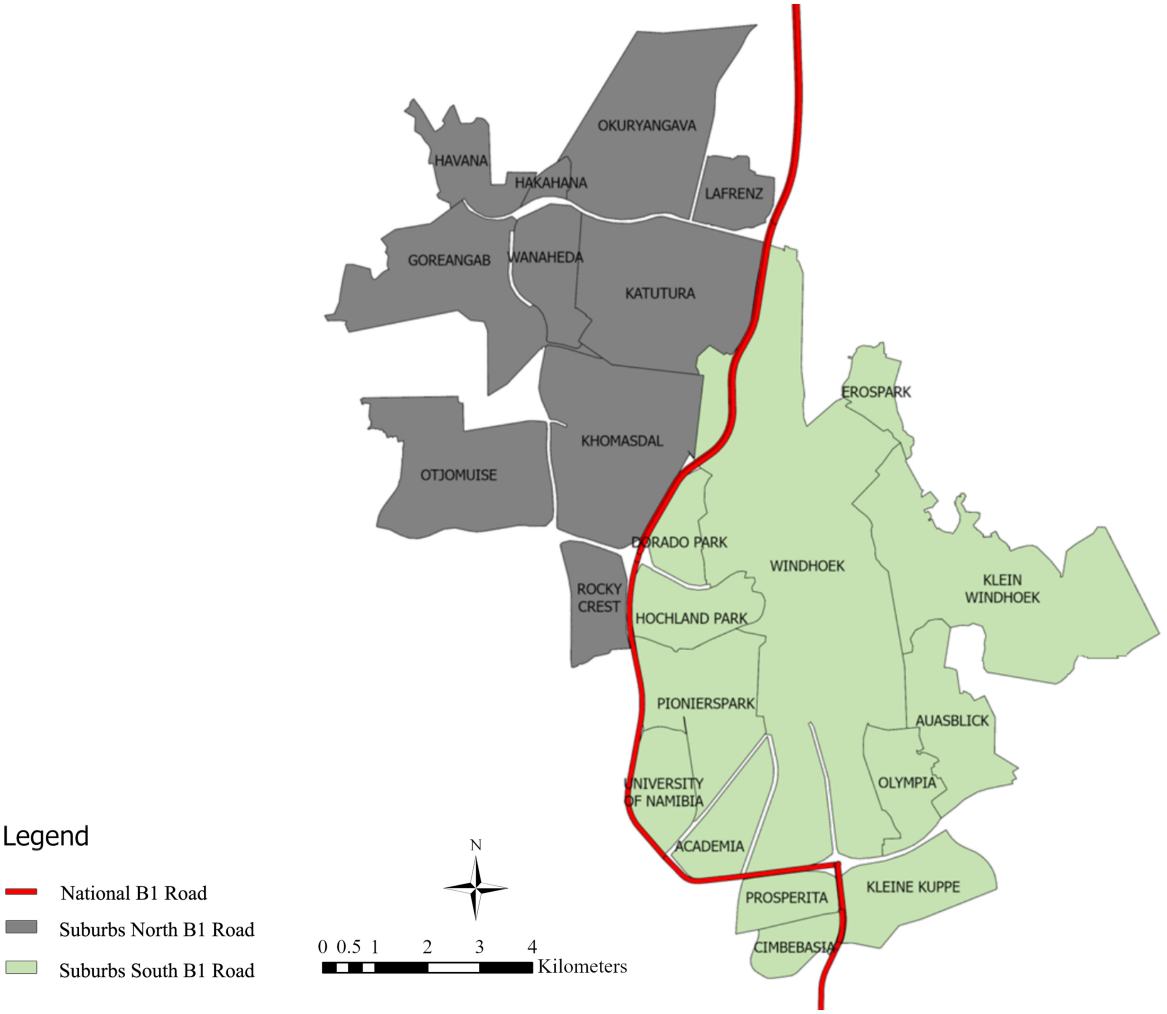
Windhoek B1 road.

This gap is especially evident in Namibia, which is rapidly urbanizing (3.70% population growth in Windhoek per year) and has placed strain on infrastructure, led to increased spatial inequalities, and heightened housing, employment, and mobility demands (7). The urban structure of Windhoek is not very closely linked with the transport system. Access to some areas is good whereas some are not. It is somehow connected with the planning during the period of apartheid that separated communities according to economic and racial factors, which further complicates urban development. For instance, the B1 road/Western Bypass is the road that separates the rich southern part and the poor northern part of Windhoek (8). As illustrated by Figure 1, the middle-income and the poor-income people live in Windhoek in the suburbs indicated by gray color. The green suburbs consist of high-income groups. The middle-low-income communities depend heavily on public transportation. The areas are however not reachable well. The high-income neighborhoods are equipped with access to the private cars, and they rarely use the public transport. Moreover, majority of the established commercial and state organizations and services are found in the south of the B1 road / Western Bypass which results in a massive spatial injustice. The situation creates the necessity to address the problems of post-apartheid-planning led urban development with the help of the new generation TOD planning approach.

A functional public transport system, along with a network of non-motorized transport, is important for enabling people to move around the city easily. In response, Windhoek’s Sustainable Urban Transport Master Plan (SUTMP) advocates TOD to mitigate congestion, emissions, as well as promote equitable access. Nevertheless, the city’s reliance on inefficient sedan taxis and car-centric mobility also highlights the urgency of TOD implementation (9). This study addresses this need by analyzing 135 bus stops using the Node-Place model, a tool that helps to assess the synergy between transport nodes and land use intensity (10).

Most of the existing literature select metro (11) or high-speed rail stations (12), and whereas less literature considers on bus stations (13). The majority of them are confined to countries or regions that have established a well-developed rail transport (14–19), and there is a lack of such studies in African countries, particularly in regions where the rail transport stations have not yet been constructed (20). Therefore, the results will contribute to TOD literature in underrepresented African contexts and provide actionable insights for Windhoek’s SUTMP, especially its planned Bus Rapid Transit (BRT) system. By providing a linkage between theory and practice, this study highlights TOD’s role in fostering compact, inclusive, and resilient urban growth in developing urban areas.

The three key contributions of the study to the TOD research are the following: First, the study gives a thorough empirical assessment of TOD performance in Windhoek in an adapted Node-Place model of bus systems. Second, it creates a replicable evaluation framework for cities of the Global South which lack city rail infrastructure. Third, it highlights the importance of the spatial equity to the TOD planning particularly in the cities with historical fragmented urban patterns. The legacy planning process in Windhoek has led to uneven distribution of transport and amenities. Addressing these disparities requires TOD strategies that go beyond the ideas of density and connectivity to involve inclusive land use and equitable provision of services.

This paper is divided into six sections. Following this introduction, Section 2 will review the theoretical foundations, which will involve the evolution of TOD principles, the node-place model framework, and the use of TOD in the context of developing countries. Section 3 details the data and methodology which includes the research framework, study area, data collection, and the selection of node-place indicators. Section 4 presents the empirical results, providing a comprehensive analysis of the node-place model results and a sensitivity analysis of the weighting schemes. Section 5 has given specific case studies of representative bus stops to exemplify the typologies that have been identified. Finally, Section 6 summarizes the main findings and discusses their implications for policy and practice. Through identifying high-performance TOD areas, this study seeks to: (1) examine the interplay between public transport and land use in Windhoek, (2) prioritize stations for TOD interventions to enhance better accessibility and equity, and (3) inform policy frameworks that reconcile the global sustainability goals (e.g., SDGs).

## 2 Literature review

### 2.1 TOD principles and evolution

Transit-Oriented Development (TOD) has changed greatly since its creation as more theories were developed together with practical concepts that were put into practice in urban planning. Originally stated by Calthorpe in The Next American Metropolis (21), TOD emerged from broader movements in New Urbanism and Smart Growth (22), which focus on compact, mixed-use development within walking distance of transit nodes. The original models were created on the basis of the 3D principles of Density, Diversity, and Design (23), which was further extended to 5D principles including Distance to transit, Destination accessibility (24), and finally graduated to the full 7D model that included Demand management and Demographic factors (25). More recently, the framework has been expanded to incorporate the 9D approach, which further incorporates the Desirability of transit, Dissonance and Deference to the environment (26), to provide a more comprehensive evaluation of TOD performance in the context of developing countries (Table 1).

**Table 1:** TOD principles evolution table.

Name	Elements	Representative references
3D	Density, diversity, design	Cervero and Kockelman (23)
4D	Density, diversity, design, distance to station	Huang et al. (55)
5D	Density, diversity, design, distance to transit, destination	Ewing and Cervero (24)
5D + N	Density, diversity, design, distance to transit, destination+ site attribute features	Xia and Zhang (56)
5D2	Differentiated density, Dockized district, delicate design, diverse destination, distributed dividends	Zhang (57)
6D	Density, diversity, design, distance, destination, demand management	Ogra and Ndebele (58)
7D	Density, diversity, design, distance to transit, destination, demand management, demographic	Wang et al. (25)
9D	Density, diversity, design, distance to transit, destination, demand management, desirability of transit, dissonance, deference to the environment	Mangu et al. (26)

The TOD has been widely accepted as a sustainable urban development policy framework across the world (27), especially in dealing with the issue of high urbanization rate like sprawl and congestion (28). The most important aspect of it, the integration of land use and transportation (29, 30) has been proven effective in creating pedestrian-friendly communities, transit-accessible communities (31). With more cities employing TOD strategies, the concept keeps evolving, with generalized principles alongside the required contextual changes (24). This continuous development highlights the TOD’s enduring relevance in both the academic and practical study in urban planning (32, 33).

### 2.2 Node-place model

The node-place (NP) model, developed by Bertolini (34), is known as a practical analytical tool specifically designed to estimate the performance of TOD by quantifying and evaluating the synergy between transport accessibility (node) and land-use integration (place), particularly in station areas (10). “Node” reflects the accessibility of transport, its connections and centrality of the network, and “place” reflects the functions of land-use, amenities, and urban vitality in the city.

**Figure 2:**
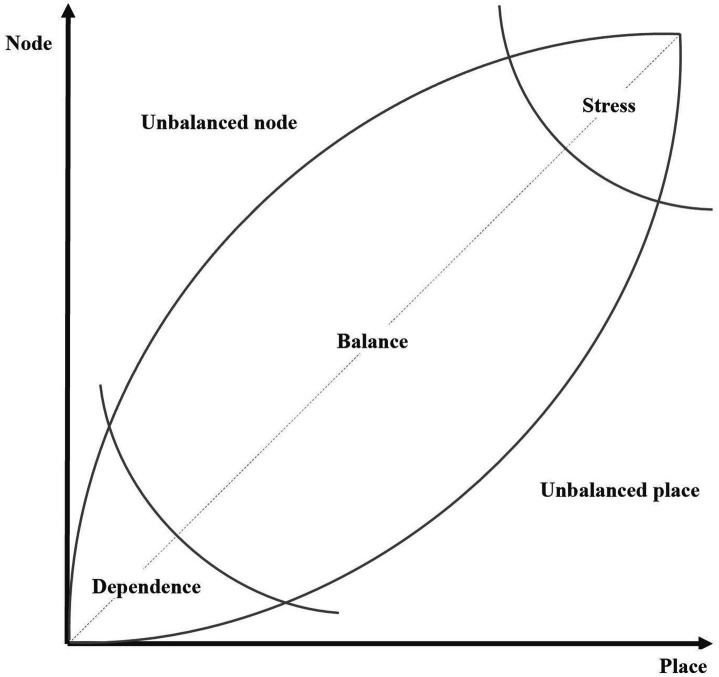
Node-place model by Bertolini (10).

Through a two-dimensional coordinate system (Figure 2), the model categorizes station areas into five typical categories, namely, Balance areas, in which transport functions and land-use activities are well-matched and synergistic; Stress areas, in which intense land-use activities may overwhelm the capacity of the transport system; Dependence areas, in which transport accessibility and land-use functions are poor; Unbalanced node areas, where transport accessibility is strong and land-use is weak and Unbalanced place areas, destinations with significant activities and amenities that are poorly served by the public transport network (10).

With deepening practical applications, the model has evolved from two-dimensional to multi-dimensional assessments (35–37): the design dimension has added metrics such as walkability (38) and urban design quality, with Sydney’s Burwood Station case which has shown that the design dimension and place function are significantly positively correlated (39). Zou and Tang (40) added an industry dimension to the Node-Place model by analyzing high-speed railway stations, thus, creating the improved Node-Place-Industry (NPI) model. Methodologically, the integration of techniques including entropy-weighted TOPSIS, space syntax (DepthMap), and K-mean clustering has significantly enhanced the scientific rigor of dynamic station evaluation and prioritization. Nonetheless, there are still significant controversies related to the use of the model. As an illustration, there is a lack of unified standards for defining spatial boundaries (using buffer zones with a radius of 500 m for BRT systems, 400 m for conventional bus stops, or 800 m for rail stations leads to inconsistent results) (41–43).

### 2.3 TOD in developing countries

As one of the key strategies used to mitigate the urban sprawl and traffic congestion, Transit-Oriented Development (TOD) has had its theoretical paradigm spread worldwide through North America, while exhibiting specific localization patterns in developing countries (32, 44, 45).

Early studies focused on the catalytic impact of rail transit on land development (46) and the TOD concept by Calthorpe further formalized the concept of transportation and land use integration (21). But third world nations simply have structural issues to implement this model: with the pressures of rapid urbanization, TOD must address several contradictions at the same time such as transport efficiency, housing shortages and organizational issues (47). East and Southeast Asian countries (e.g., China and Malaysia) increase the public transport share by high-density development (27), whereas Middle Eastern nations (e.g., Saudi Arabia, UAE) are interested in applying TOD to new town development, establishing practice directions distinct from Western models (48, 49). But African cities, such as Windhoek, face their own special challenges, including informality and land fragmentation, Apartheid-era spatial divides and bus-dependent mobility.

The implementation effectiveness of TOD in developing countries is constrained by institutional settings and deep-seated social equity issues. In the case of Jakarta Metropolitan Area, fragmented land ownership and interdepartmental coordination failures results in inefficient development around stations (50). More importantly, the social exclusion risks arise: the soaring housing costs have forced low-income earners out of the rail stations areas, a process that economists refer to as residential displacement effect, and the lack of affordable houses in Malaysia has further compromised the spatial equity of the vulnerable groups (51). It is worth noting that rail transit’s substitution effect is often overestimated—Cervero’s empirical research shows that decreased use of the private cars hinges more on increased housing density and parking restrictions rather than the proximity to the stations (23), which have been ignored in the planning of developing countries.

In order to address these issues, researchers suggest localized innovation paths. Methodologically, China has developed a multi-scale city-corridor-station planning, land value capture is used to reinvest on the public transport. Institutionally, the Southeast Asian countries systematize Sustainable Development Goals (SDGs), including the affordable housing ratios and non-motorized transport connection into TOD assessment systems (52). These practices represent a paradigm shift in the theory of TOD to institutional flexibility. Nevertheless, there are still significant research gaps: empirical studies are still mostly based on developed nations, and there are few interdisciplinary approaches to methodologies. Future work should focus more on the quantitative evaluation of TOD to promote the tool as a potent solution to sustainable urbanization in the Global South.

In summary, the existing literature on TOD and node-place model indicates the presence of three critical gaps. First, whereas much has been done to understand rail-based TOD in developed areas, conventional public transport infrastructures, in particular, bus networks in the African cities such as Windhoek, remain understudied, despite their importance to urban mobility in resource-constrained contexts. Second, the current frameworks often ignore the structural peculiarities of fast-urbanizing cities of the Global South, such as spatial discrimination of apartheid-era planning in Windhoek, which require specific adaptations of the TOD principles (44, 45). Third, there are methodological inconsistencies [especially setting the spatial limits (e.g., buffer radii)] and weighting indicators, which restrict cross-case comparability.

## 3 Data and methodology

### 3.1 Research framework

**Figure 3:**
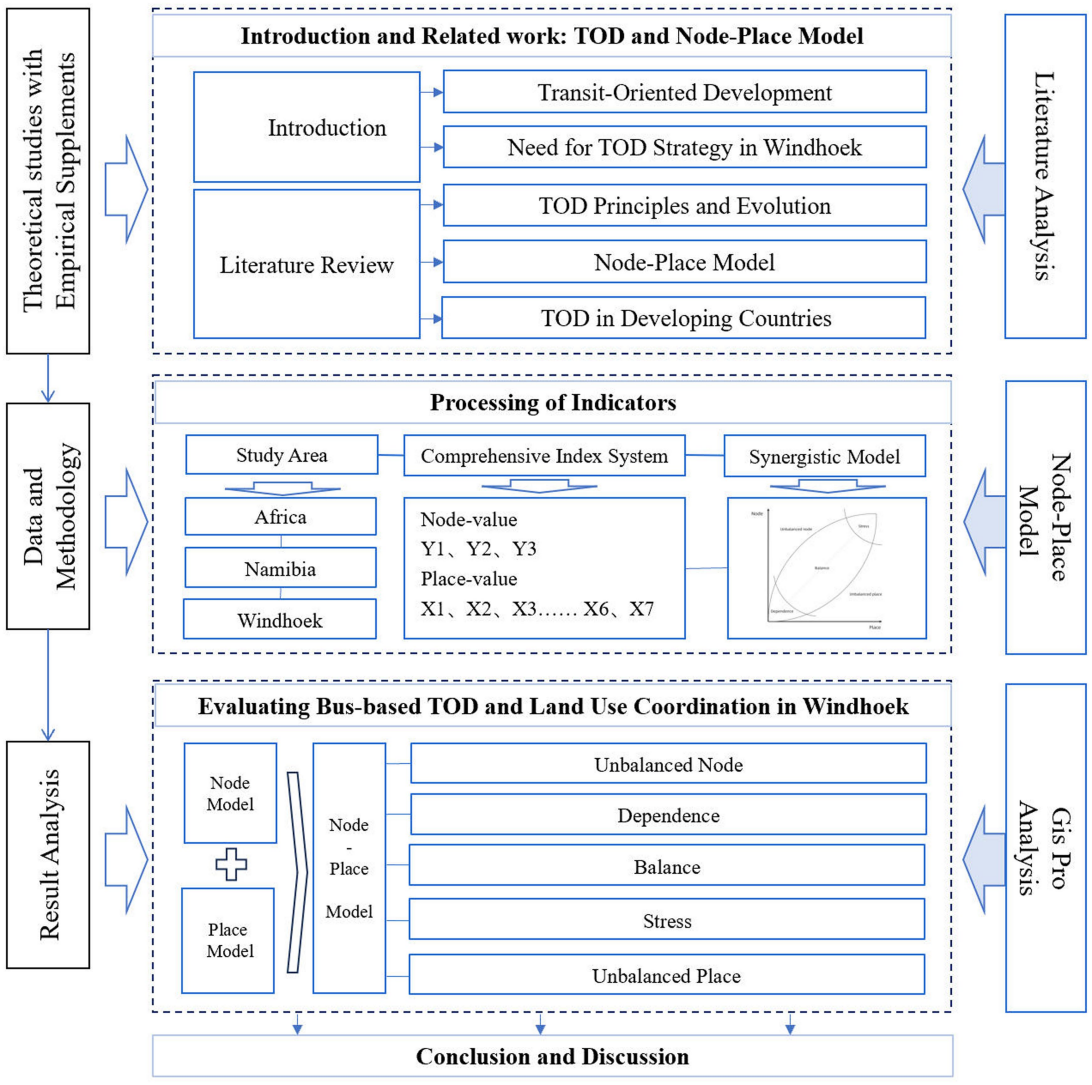
Research framework.

Based on the Node-Place model, this research uses the model to process a systematic assessment of the performance of Transit-Oriented Development (TOD) based at the bus stop level in Windhoek by operationalizing the assessment using three node indicators and seven place indicators within a 400-meter buffer zone of each bus stop. The methodology combines the geographic information system (GIS) analysis through ArcGIS Pro 3.0 to process and visualize spatial data, and all data collection and analyses are restricted to the period 2023–2024 to ensure consistency in cross-sectional comparisons. The research framework is as follows (Figure 3).

### 3.2 Study area

**Figure 4:**
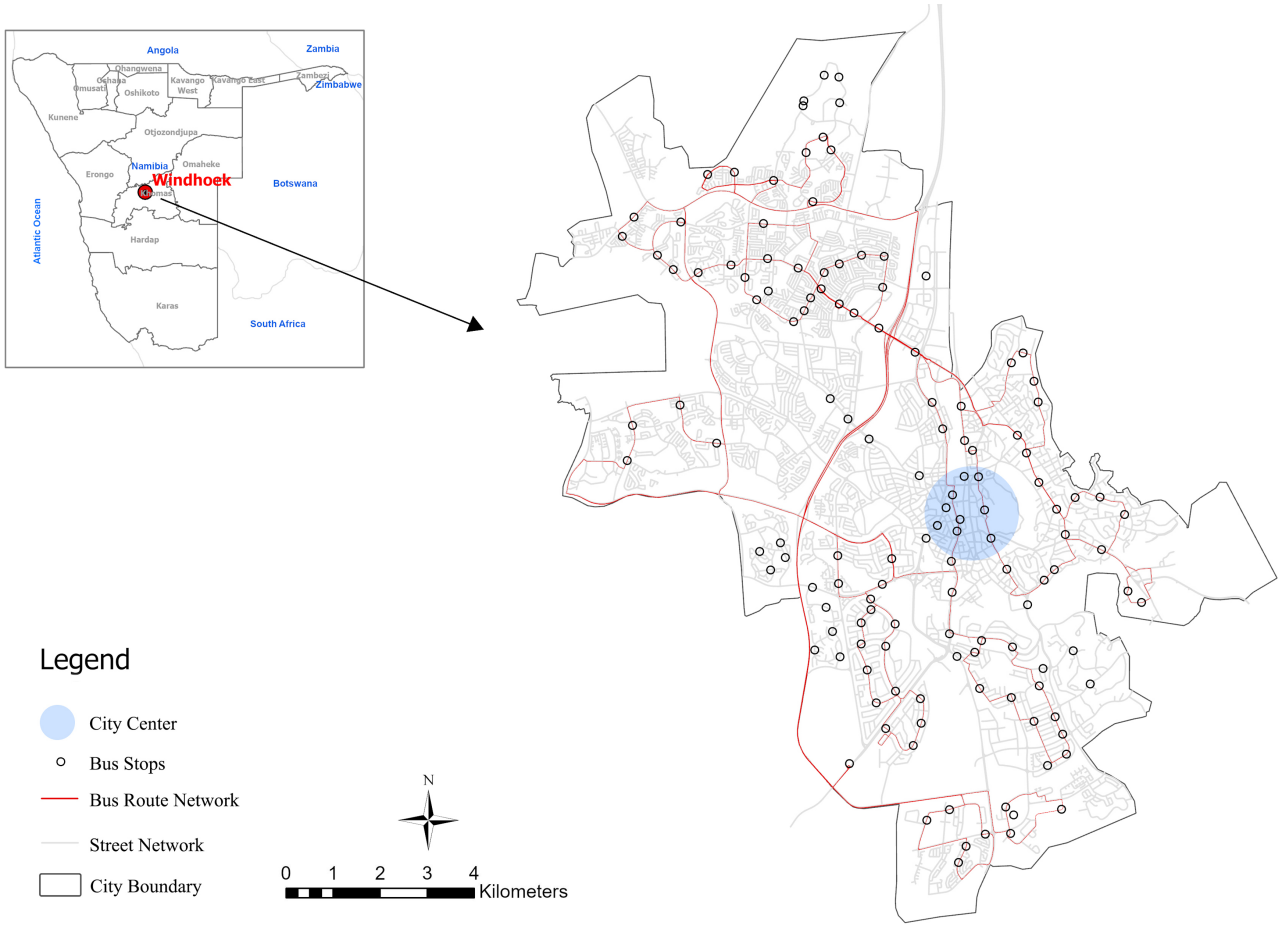
Study area in Windhoek city.

The study area is Windhoek, the capital and largest Namibian city which occupies a central geographic position taking a total land area of about 5,133 km^2^ (Figure 4). Since the establishment of the German colonies in Namibia in 1890, Windhoek has developed to become the economic, administrative, and educational center of the country, with a population of over 400,000 people now or over 15 percent of Namibia’s population of 3 million. The urbanization of the city is swift with a population growth rate of 4% per annum and the informal settlements also have a higher rate of growth at 10% per annum indicating unequal distribution of basic service provision. The urban evolution of Windhoek has been significantly influenced by the colonial history especially in the policies that employed the spatial planning during the apartheid rule that created persistent patterns of racial segregation and automobile-dependent mobility.

### 3.3 Data collection

To conduct this study for the Windhoek bus station area, a wide variety of data on land use, building and transportation was needed. Multisource open urban data were chosen to construct the indicator systems used as features in the Node-Place Model to create a TOD typology of the Windhoek bus stops. Land use data (2022) was obtained from the City of Windhoek’s Urban and Transportation Planning Department. The data was cleaned and the zonings were reclassified to allow for a more manageable set of land uses as the initial land use zonings are too many, this study reclassified them to 6 land use zonings (Table 2). Geometries were also corrected and rectified in ArcGIS Pro Version 3.0.

**Table 2:** Land use data renaming table.

Original name	Rename
Industrial	Industrial
Residential	Residential
General residential	
Business garage	Office, business, institutions, special
Institutional	
Office	
Restricted business	
Special	
Farm	
Government	Public administration
Municipal Undetermined	
Cemetery	
Problem	
Street	Streets, transport, communication
Transport	
Communication	
Private open space	Open space
Public open	
Space	

The building footprint and floor data were obtained from geofabrik.de (https://download.geofabrik.de/africa/namibia.html, accessed on October 16, 2023). Geofabrik provides free geodata created by projects like OpenStreetMap. The data underwent geometric repair and topological cleaning procedures before calculating average floor area ratios (AFAR) for 400 m × 400 m grid cells across Windhoek. For the node-place model analysis, transportation datasets – including street networks, bus stops, bus routes, and taxi rank locations – were acquired from Windhoek’s Urban and Transportation Planning Department and subsequently processed through topology validation and network analysis in ArcGIS Pro. All data were collected from October 2023 to February 2024.

### 3.4 Indicators and weights

This study adapts the node-place model to Windhoek’s car-dominated mobility context, where conventional busses (without BRT or rail systems) serve as the sole formal public transport option. Accordingly, we employ an adapted version of the standard node-place model of 10 measures (3 node indexes and 7 place indexes) to evaluate accessibility and activity intensity at bus stop, utilizing a 400-meter pedestrian catchment area based on the typical walking tolerance for bus-based systems in developing cities (43) (Table 3).

**Table 3:** Selected node-place model indicators.

Values	Indicators	Measurement	Weight
Node-value	Number of stations within a 400 m radius (Y1)	Number of stations reachable within a 400 m radius to bus stop (index stop excluded)	0.30
	Proximity to CBD (Y2)	Accessibility to CBD (operationalized as proximity inverted after normalization)	0.45
	Number of directions served by bus stops (Y3)	The total count of distinct bus routes serving the stop	0.25
Place-value	Industrial land (X1)	The area of industrial land within 400 m walking distance from bus stops (D1)	0.10
	Residential land (X2)	The area of residential land within 400 m walking distance from bus stops (D2)	0.15
	Offices, business, institutions, special land (X3)	The area of offices, business, institutions, special land within 400 m walking distance from bus stops (D3)	0.20
	Public administration land (X4)	The area of public administration land within 400 m walking distance from bus stops (D4)	0.10
	Streets, transport, communications land (X5)	The area of streets, transportation, communications land within 400 m walking distance from bus stops (D5)	0.15
	Open Space land (X6)	The area of open space land within 400 m walking distance from bus stops (D6)	0.10
	Degree of Functional Mix (X7)	$\mathrm{Mix}=-\frac{\sum_{k=1}^{n}(P_{k}\times \ln P_{k})}{\ln k}$ , where $P_{k}$ is the share of land-use class k within 400 m and *n* is the number of present classes.	0.20

The choice of the indicators is based on the TOD 5D principles (24) and has been adjusted according to the dynamics of bus-based TOD in the African cities. To quantify accessibility in each node (the node-value), this study used three indicators of connectivity in the local urban structure of Windhoek: number of stations within 400 m radius (Y1), which tailors the assessment of accessibility to the local dynamic of relying on the density of the network, as opposed to its proximity to one station; travel time to the CBD (Y2), which is an assessment based on the Destination dimension, and is specific to the dynamics of the monocentric urban form in the city of Windhoek; and he number of distinct bus routes served (Y3), a metric tailored to capture the dynamic of travel diversity and connectivity patterns within African cities (13).

Seven land-use indicators were used to measure the “Place value: the indicators were chosen and modified to fit the context of the city of Windhoek based on existing TOD models. Many cities in Sub-Saharan Africa have a centralized employment sector in particular regions and residential suburbs would tend to be monofunctional. Thus, such indicators like the existence of offices, the administration of the population, and residential land are crucial in determining the diversity of land-use. Moreover, the requirement of public open space to the population cannot be ignored, since it performs significant social and economic roles in the life of African cities. We started by determining the geometric area of the land use type of each bus stop (industrial, residential, offices, public administration, transportation and public open space) within the 400-meter buffer of each bus stop. The entropy weight method was used to then quantify the functional mix (53).

All raw indicator values were initially scaled to a 0–1 range using min–max scaling in order to allow them to be compared and aggregated across the various node and place indicators. For the cost-type indicator Y2 (travel time to CBD), the values were inverted following normalization whereby larger values always indicate good performance. The composite indices are built using three weighting regimes AHP weights (Table 3) based on the expert elicitation as local policy priorities; entropy weights based on data dispersion to objectively represent variability of the indicators; and equal weights as a transparent benchmark. Our main specification of the policy-oriented discussion is the AHP-weighted NP index, and robustness tests are performed on the entropy and equal weights. Section 4.3 gives the comparative analysis of all the three schemes.

## 4 Empirical results

### 4.1 The node-place model application for Windhoek

**Figure 5:**
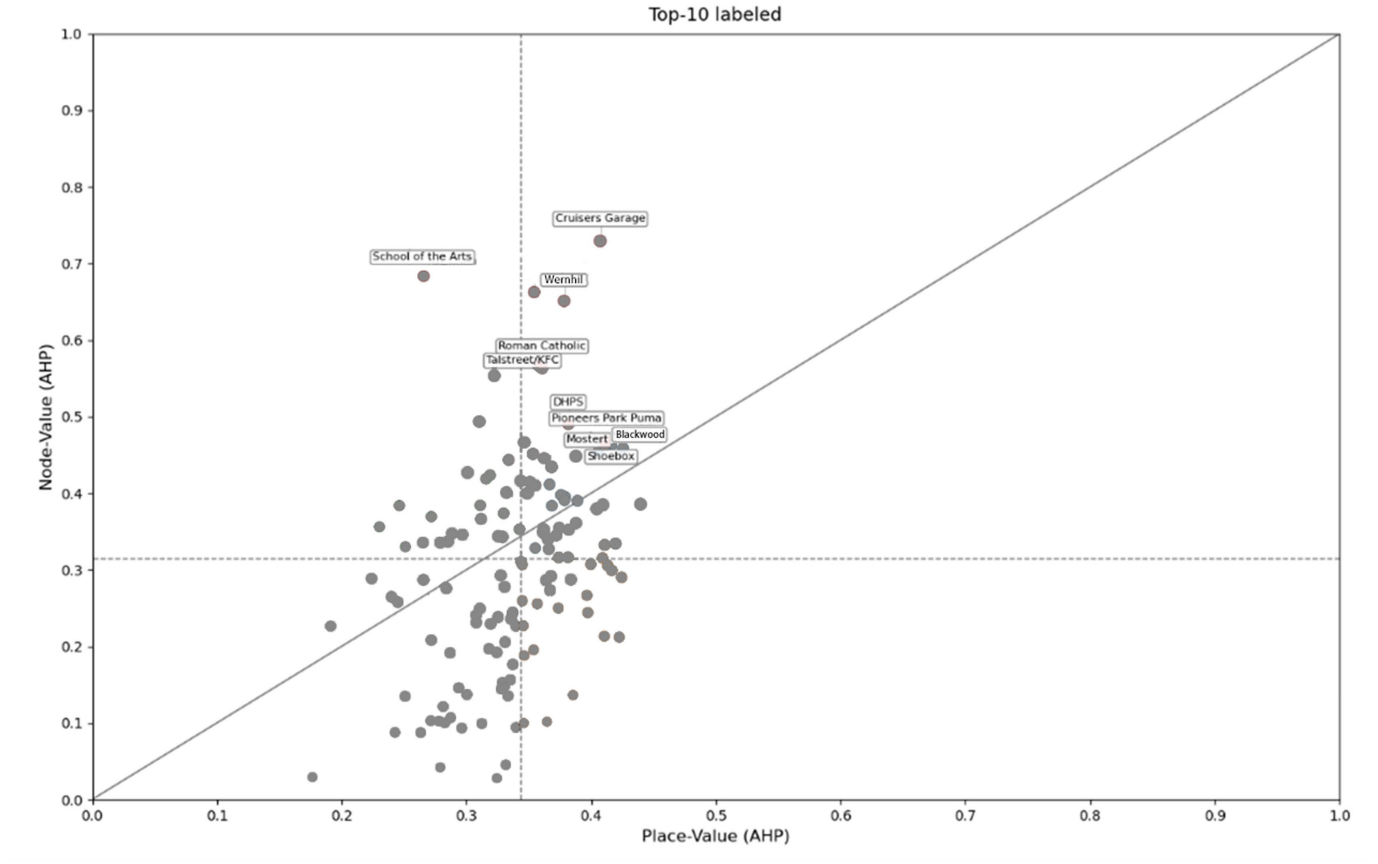
The 135 existing TODs identified by the node-place model.

The Figure 5 displays the node-place Index results of the 135 bus stop areas in Windhoek plotted on a node-place graph. It is found that the vast majority of bus stops has a place index higher than node index (80 out of 135 bus stops), suggesting prevalent areas where land-use intensity exceeds transport accessibility. Notably, bus stops with strong TOD performance (high node index and high place index) are mainly concentrated in the central business district, whereas the unbalanced conditions—especially the node-deficient cases—are widespread around peripheral suburbs. Notably, no stress areas (characterized by both high node and place indices) were identified among the studied bus stops. The distribution reflects context-specific challenges in Windhoek, such as car-dominated mobility and historical spatial inequities.

### 4.2 Spatial distribution characteristics

**Figure 6:**
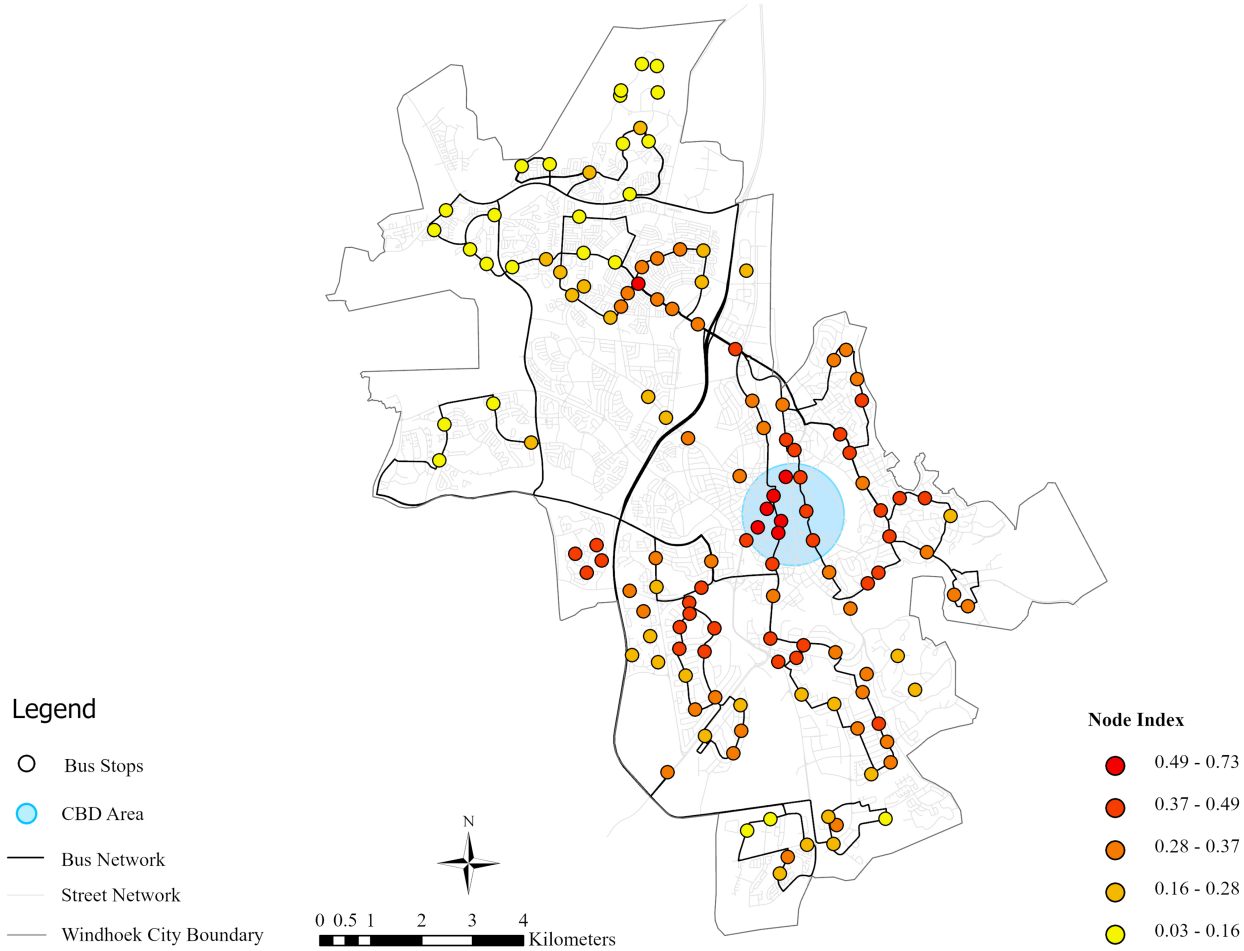
Bus stop node-value index in Windhoek city.

The Node-value calculations for the 135 bus stops reveal a range from 0.03 to 0.73, with a mean of 0.30 and a standard deviation of 0.14. As illustrated in Figure 6, high node values are strongly concentrated in the city center, reflecting superior transport accessibility and marking these zones as priority areas for TOD-oriented densification. By comparison, northern areas display consistently lower values, with the vast majority of stops falling below the mean—indicating underdeveloped public transport networks that need service improvements and infrastructure investment. The spatial mismatch between central accessibility and peripheral deficiency also raises equity concerns, as the lower-income northern communities have restricted access to city opportunities. Addressing this through targeted service expansion and equitable transit planning is essential for inclusive urban development.

**Figure 7:**
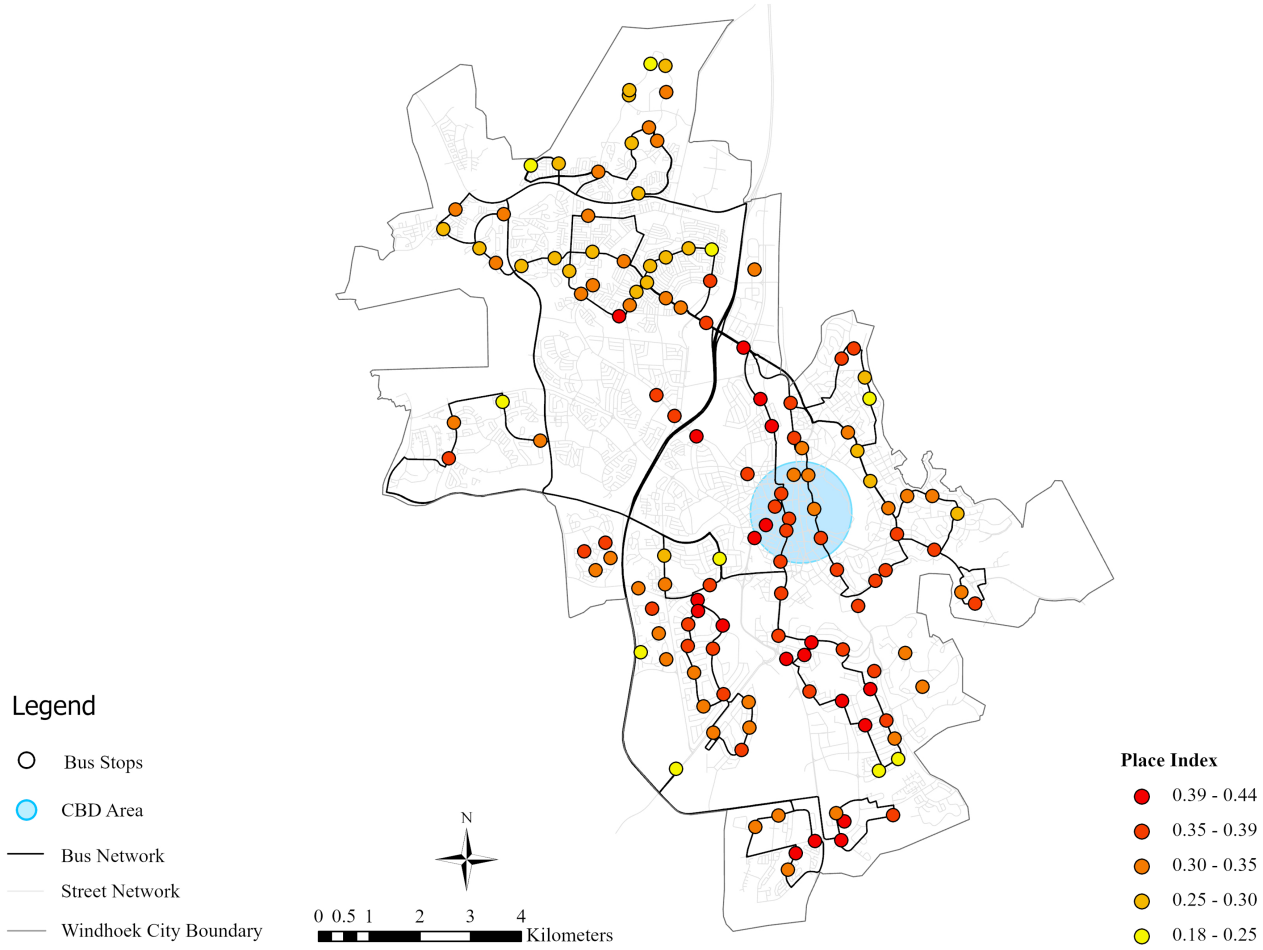
Bus stop place-value index in Windhoek.

The descriptive statistics for the Place-value Index show a range from 0.18 to 0.44 across the 135 bus stops, with the mean and standard deviation of 0.34 and 0.05, respectively. As Figure 7 shows, high-place-value areas are pre-dominantly located in the business central business district (CBD) and the southern side of the city which is south of the B1 road. On the contrary, the low-value areas are widely spread across the northern suburbs, which is indicative of a distinct spatial dichotomy formed by the historical urban planning. The southern region is characterized by affluent neighborhoods with concentrated commercial, administrative, and public service amenities. The northern area, however, is predominantly dominated with middle- to low-income communities which have limited access to urban services. The place-value index demonstrates the existing spatial inequity, which highlights the necessity of specific TOD interventions.

**Figure 8:**
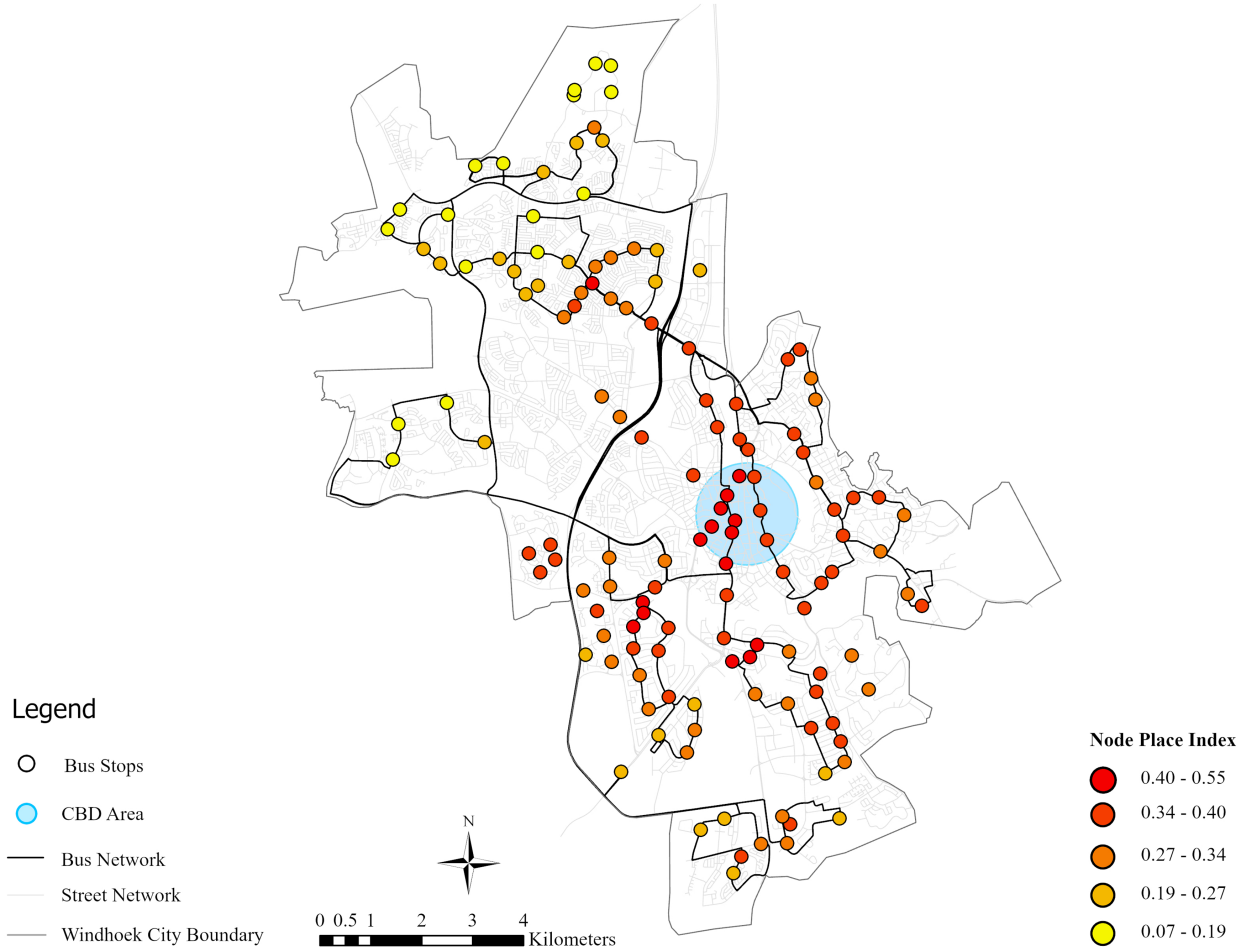
Bus stop node-place value index in Windhoek.

The node-place index is the geometric mean of node index and place index. The lowest value is 0.07, the highest value is 0.55, the mean one is 0.31 and the standard deviation is 0.09. Its spatial distribution of the node-place index in Windhoek exhibits a pronounced core-periphery structure, characterized by a strong concentration of high-value nodes in the central business district where both transport accessibility and land-use diversity are high, forming the city’s primary TOD core (Figure 8). This central agglomeration is complemented by a pattern of corridor extension along major transport arteries to the south and northeast, where medium-high values indicate potential developmental axes, while isolated secondary nodes in northern and southern areas function as local sub-centers yet remain poorly integrated into a cohesive network. Most significantly, extensive low-value areas across the urban periphery disclose a predominant state of dependence characterized by simultaneous lack of both public transport service and land-use functionality, a spatial inequality which has the roots in the planning tradition of the apartheid period. Such an explicit spatial hierarchy requires varied planning interventions: the quality improvement and congestion management in the high-value core, developing strategic corridors in medium-value axes, and ensuring the main improvements in the low-value peripheral areas to overcome historical spatial inequities through targeted transit upgrades and land-use diversification.

### 4.3 Sensitivity analysis of weighting schemes

In order to make sure that our findings are robust and to estimate the sensitivity of the Node-Place (NP) Index to various methodological assumptions, we have performed a thorough analysis of the three weighting schemes: the primary weights that are used in the Analytic Hierarchy Process (AHP) (reflecting local expert priorities), the entropy weights (dependent on the data dispersion), and the equal weights (considered a baseline of the neutral analysis).

It is found that ranking stability is very high between AHP and equal weighting (Spearman’s *ρ* = 0.988), and moderate–high between AHP and entropy (*ρ* = 0.746) and entropy and equal (ρ = 0.789). Priority lists are correspondingly stable at the top of the distribution: Top-10 overlap is 8/10 (AHP–Equal), 4/10 (AHP–Entropy), and 6/10 (Entropy–Equal) (Table 4); Top-15 overlap is 13/15, 9/15, and 9/15, respectively. However, it is imperative to mention that this central category of the high-performing TOD sites (e.g., Cruisers Garage, Wernhil, School of the Arts) is always found in all approaches, which proves the robustness of the central places.

**Table 4:** Node-place ranks and values for Top-10 (AHP) with entropy and equal-weight comparison.

Bus stop	NP index			Rank		
	AHP	Equal	Entropy	AHP	Equal	Entropy
Cruisers Garage	0.545	0.471	0.151	1	1	7
Wernhil	0.496	0.417	0.139	2	2	8
School of the Arts	0.484	0.407	0.137	3	4	9
Roman Catholic	0.451	0.376	0.119	4	11	14
Talstreet/KFC	0.450	0.374	0.112	5	12	18
Blackwood	0.442	0.382	0.124	6	10	12
Pioneers Park Puma	0.440	0.395	0.118	7	5	15
Shoebox	0.439	0.385	0.118	8	7	16
Mostert	0.436	0.383	0.111	9	9	20
DHPS	0.433	0.383	0.232	10	8	4

This difference can be mainly explained by the essential difference in the methodological approach of the entropy approach. Entropy weighting is a purely data-driven method, where indicators with larger inherent variability in the dataset are weighted, based on the idea that the larger the variance, the larger the informational content. This in our study led to a disproportionately high weight of 0.61 on the Industrial land (X1) indicator. Conceptually, as far as policy and planning is concerned, we find this outcome problematic. It means that the existence of industrial land is overwhelmingly considered the determinant of the TOD performance of a bus stop area.

As such, we chose AHP-weighted NP index as our main headline specification. This is justified as it combines the sophisticated understanding and strategic objectives of the professional, such that the outcomes are relevant in the context of the urban planning issues of Windhoek and does not create the conceptual trap of the entropy approach which focuses too much on statistical variance over the practicality of planning.

## 5 Case studies

To provide an in-depth analysis of the node-place characteristics of Windhoek’s bus stops, this study selects two representative cases that will be studied in detail. The first case focuses on the Roman Catholic bus stop, which ranks fourth according to the AHP approach, representing a well-balanced TOD area. The second case examines the Miami Service Bus Stop, which is ranked twelfth according to AHP framework, as an example of an unbalanced node area within the node-place classification system.

### 5.1 Analysis of balanced areas: Roman Catholic bus stop TOD area

**Figure 9:**
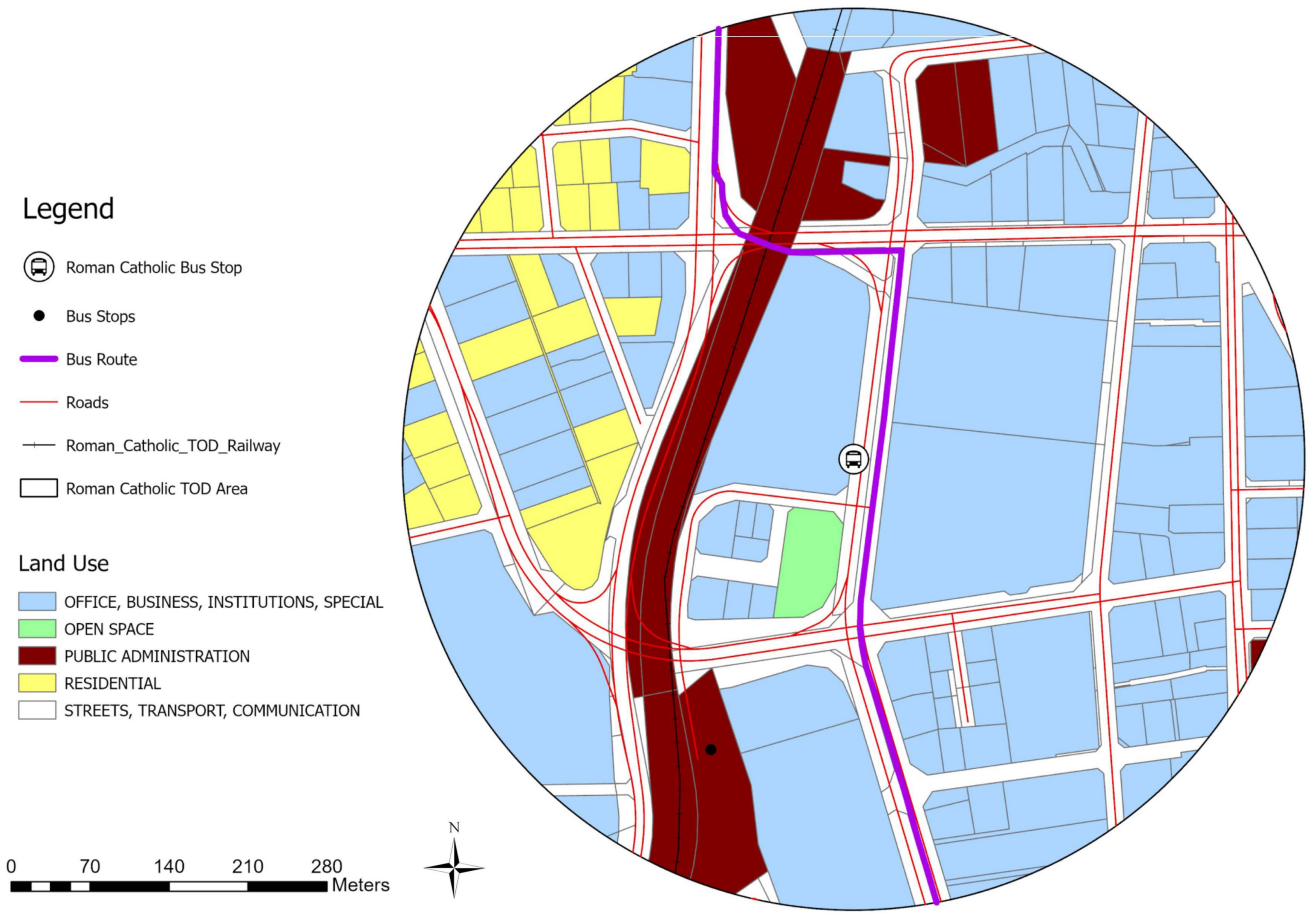
Roman Catholic bus stop TOD area (400 m buffer).

These are bus stop areas with Node-Place Index values showing a balanced relationship between transport and land use. The Roman Catholic case study has been selected to represent a balanced TOD area, as represented in Figures 8, 9 as discussed below. The Roman Catholic bus stop TOD area is fourth in Node-Place Index value (0.32) (Table 3). The most prominent are office, business and institutional land, occupying 278,549 m^2^ representing 55.4% and then the streets, transport and communication infrastructure which occupies 132, 834 m^2^ (26.4%). The percentage of public administration is 54,897 m^2^ (10.9%), residential land is 31,652 m^2^ (6.3%) and the percentage of the open space is minimum at 4,565 m^2^ (0.9%) (Figure 9).

**Figure 10:**
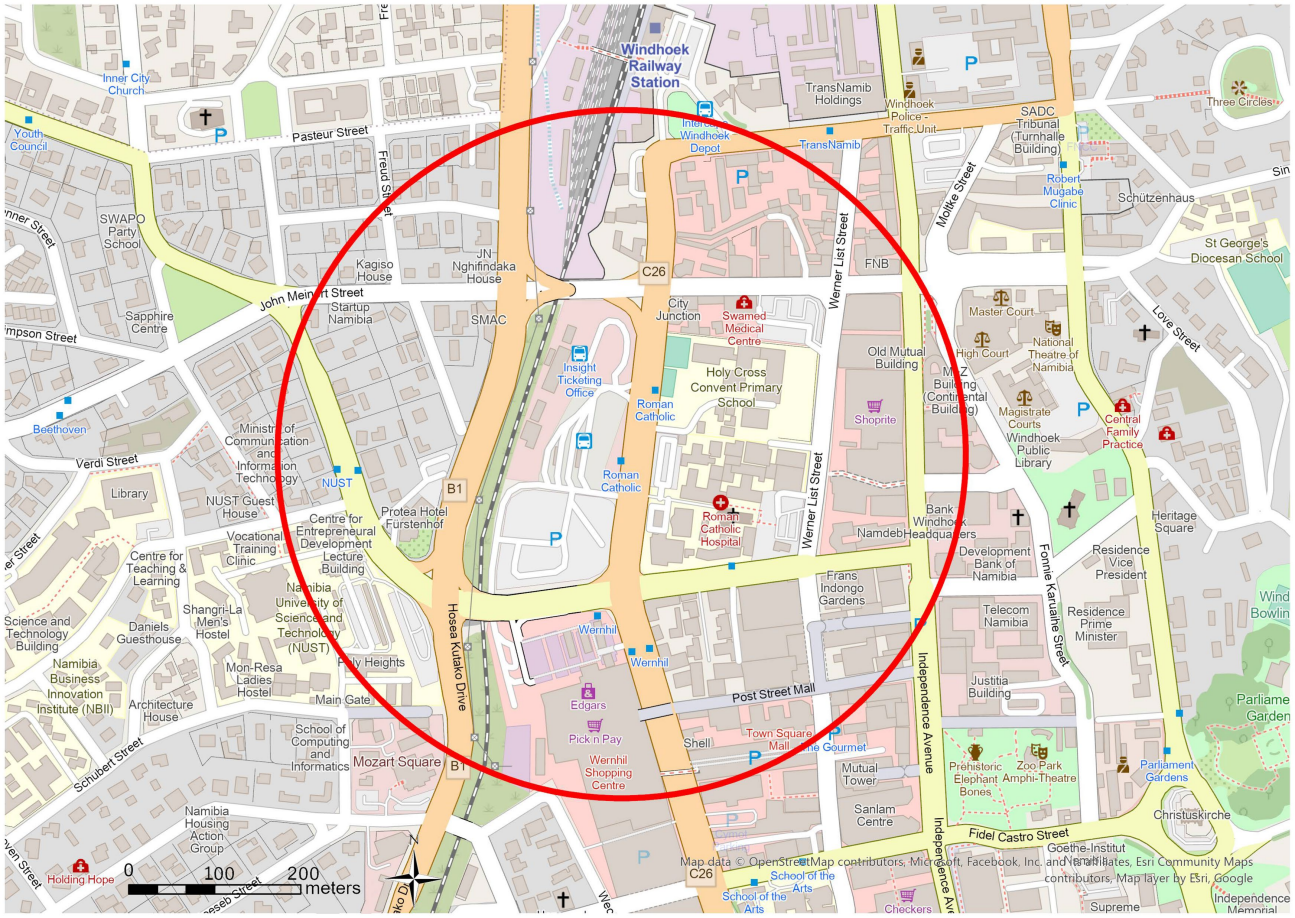
Open street map of Roman Catholic bus stop TOD area.

Located near the TransNamib railway line and central civic amenities, the area demonstrates a well-integrated mix of employment, public services, and access to transport. Although the railway does not serve intra-city transit, its presence reinforces the area’s strategic importance. Figure 10 indicates the spatial plan of the TOD buffer based on the OpenStreetMap data. To sustain this balanced development, some policy levers that should be considered are protecting the employment density by zoning of the area where the current office and institutional land uses are preserved, and zebra crossing and curb radii minimization as pedestrian safety measures should be taken to provide more walkability and balanced nature of the area.

### 5.2 Analysis of unbalanced node areas: Miami service bus stop TOD area

**Figure 11:**
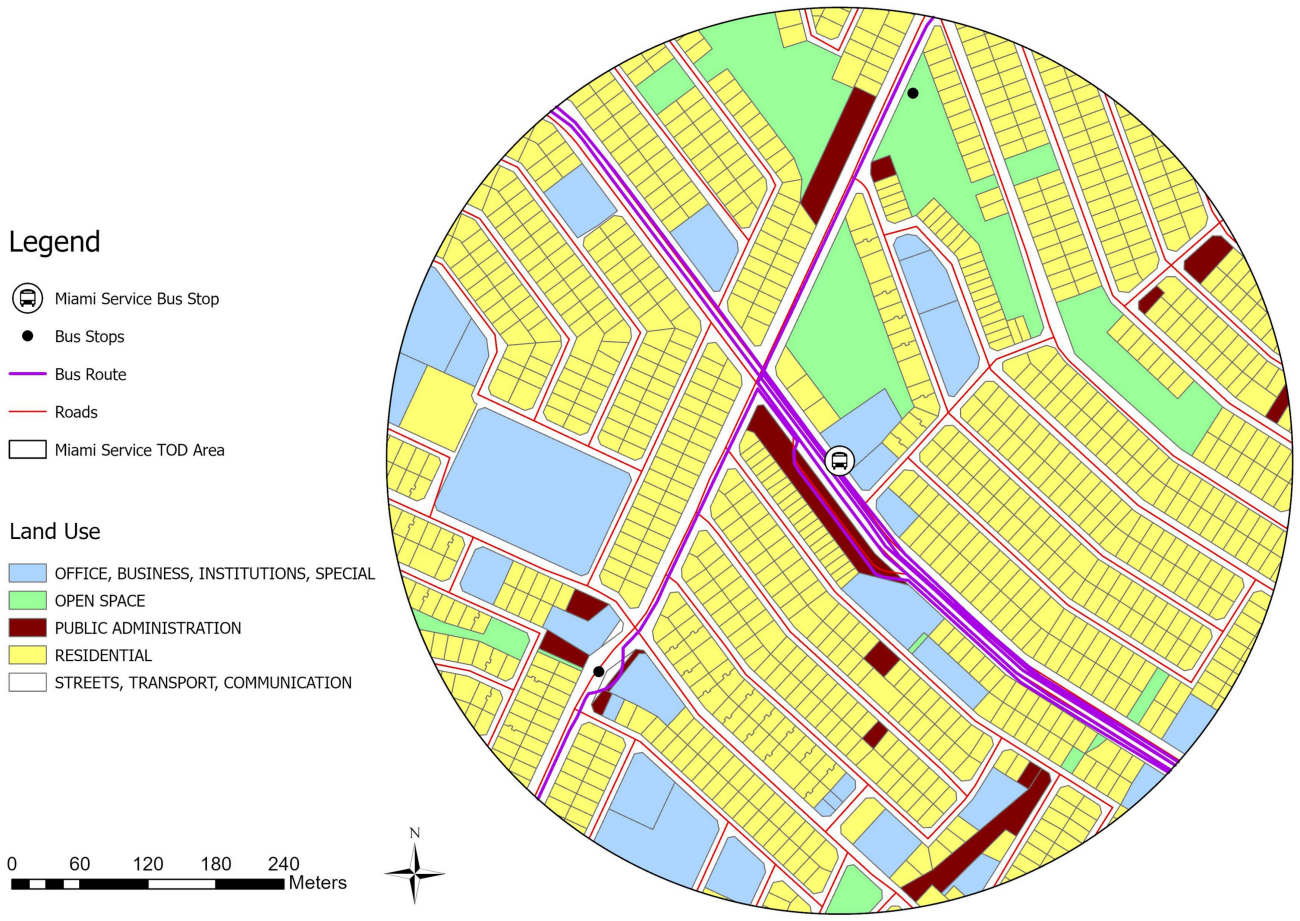
Miami service TOD Area (400 m buffer).

**Figure 12:**
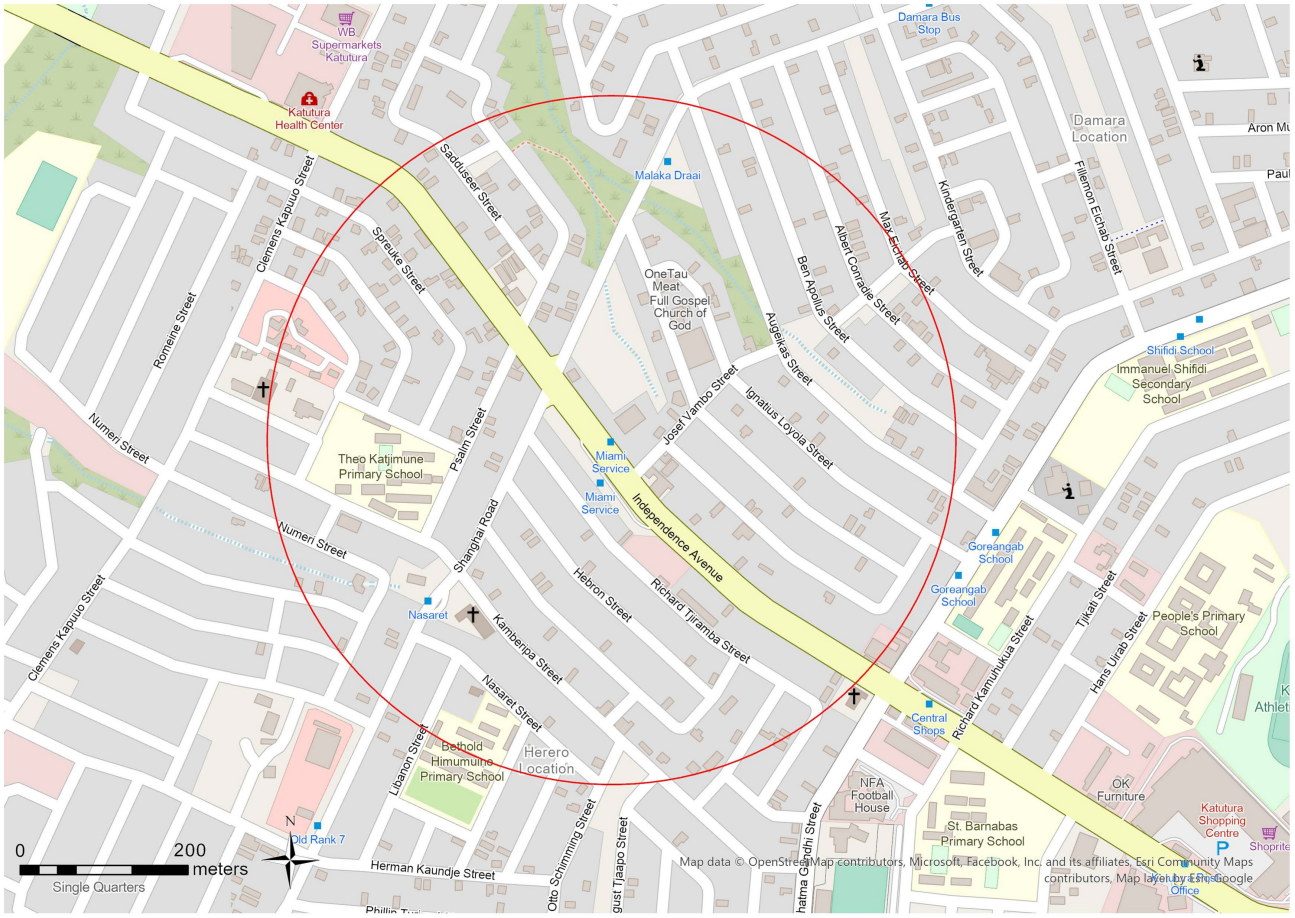
Open street map of Miami bus stop TOD area.

Unbalanced nodes are those bus stop areas with node-place index values indicating a bias toward transportation infrastructure or land use. We chose Miami Service as case study, which has a notable disparity between its node index (0.683) and place index (0.266), thus it is considered to be an unbalanced node area in the framework of node place model. The Miami Service Bus Stop area, with the second-highest Node-Place Index value of 0.37 in the Windhoek City, exhibits distinct land use characteristics within its vicinity (Figures 11, 12). Notably, the area encompasses various land use categories, including residential land which dominates, with the area of 262,784 m^2^ (52.3%), followed by streets and transport infrastructure at 121,319 m^2^ (24.1%). Office, business, and institutional land accounts for 60,951 m^2^ (12.1%), whereas open space and public administration together constitute less than 12%.

The bus stop is located along Independence Avenue, which is a major north–south arterial corridor in Windhoek. Its strategic location provides high accessibility to transport, but the land use of the surroundings is biased toward housing. Other nearby bus stops, like Malaka Draai and Old Rank 7, serve to add to the area’s spatial dynamics but without remarkable impact on its functional imbalance. In Figure 11, the spatial layout of the Miami Service TOD area is depicted by means of OpenStreetMap data. The strong transport access, the dominance of residential land and limited civic or commercial presence reinforce Miami Service’s classification as an unbalanced node. Targeted zoning reforms and land use diversification are needed to support TOD principles and improve the area’s long-term development potential.

### 5.3 Areas under stress and dependence

Among the bus stops studied, stress areas, experiencing stress due to high demand on transportation systems or land use, were not identified, indicating no current congestion risks at these nodes. In contrast, the majority of bus stops exhibit dependence characteristics, which are found to have low development levels. Such dependence areas present both challenges and opportunities, whereas, currently, there is poor walkability and lack of diversity of land use, the existing transport accessibility offers a basis of targeted TOD interventions.

The absence of stress areas combined with widespread dependence conditions point to the fact that Windhoek’s urban development has not yet reached the congestion thresholds seen in more mature transit cities, while at the same time demonstrating substantial potential for coordinated transport and land use improvements. This pattern particularly highlights the opportunity to introduce phased TOD strategies in dependence areas, emphasizing on increasing density, enhancing mixed-use development, and augmenting pedestrian connectivity around existing transit nodes to lessen over-reliance on transportation infrastructure while taking advantage of established accessibility.

## 6 Conclusion and discussion

This study offers a comprehensive assessment of Transit-Oriented Development (TOD) performance in Windhoek, Namibia, using the node-place model with Analytic Hierarchy Process (AHP) approach, which is complemented by entropy weighting and equal weighting approaches for sensitivity analysis, to evaluate 135 bus stops. The findings indicate that the vast majority of bus stops (80 out of 135) exhibit a place index higher than node index, indicating prevalent areas where land-use intensity exceeds transport accessibility. The concentration of high-performing TOD nodes in the central business district contrasts sharply with the widespread “dependence” category characteristics found in peripheral areas. The spatial patterns show a stark contrast between well-connected nodes and underserved areas, indicating the lingering effects of apartheid-era planning. The low Node-Place scores in peripheral suburbs can be directly explained by historic under-investment in infrastructure and services, which necessitates an equity-first approach for TOD upgrades. The absence of “stress” areas suggests Windhoek has a unique opportunity for proactive planning before congestion patterns become entrenched.

The results are in line with broader challenges in African urban development, where TOD implementation must address historical spatial inequalities while fostering sustainable mobility. As we examine, the results reveal how Windhoek’s B1 road creates a distinct socioeconomic divide, with northern communities facing limited access to opportunities despite heavy reliance on public transport. To address these challenges, our findings offer local planning authorities a diagnostic tool for targeted interventions by providing a “diagnosis-and-prescription” tool. For “Dependence” areas that are predominantly located in northern part of the city, the policy should focus on foundational investments, i.e., tactical urbanism (e.g., pop-up markets, parklets) in order to activate underutilized spaces, encouraging infill development on public land for clinics or community facilities, and implementing comprehensive pedestrian infrastructure improvements, i.e., continuous shaded walkways, enhanced lighting, and safe crossings. For “Unbalanced Node” areas, strategies should involve exploiting existing transit infrastructure by mixed-use overlay zones and targeted up-zoning to increase commercial density. In the case of “Unbalanced Place” areas, the strategy must consider the enhancement of transit services through increased frequency and optimization of routes. Lastly, in the case of “Balanced” areas, the policy should be directed toward preservation and enhancement.

Nonetheless, these suggestions present practical challenges in the context of the country of Windhoek, which has a distinct political-economic setting. The institutional fragmentation of the city, strained municipal fiscal situations and complicated historical land tenure problems pose obstacle to the implementation of mixed-use zoning incentives and equity-based investments. Success, therefore, will depend not only on technical planning but also on strong political will, innovative financing mechanisms, and committed cross-departmental collaboration.

Importantly, these interventions must be sensitive Windhoek’s urban history, so that TOD planning should be active to redress historical inequities instead of reinforcing them. In fact, our proposed framework does not only address spatial inequalities but also delivers significant public health co-benefits by providing key social determinants of health. In our analysis, we identifies “dependence” areas, concentrated in the northern suburbs and characterized by poor access to transport and amenities, as priority zones for such integrated interventions. In this case, TOD can directly reduce health disparities in the following ways: First, by enhancing node-place synergy to enhance walking exposure, which would enhance physical activity and lower risks of non-communicable diseases such as obesity. To make this active travel culture, it is important to transform not-so-balanced nodes into vibrant mixed-use areas. Second, through the enhancement of bus service to trigger a transition to public transit instead of the individual car, which reduces air and noise pollution in the locality, which are risk factors known to cause respiratory and cardiovascular diseases. Third, establishing balanced node-place space and upgrading dependence stops to enhance direct access to essential health facilities such as clinics and pharmacies, therefore, filling critical gaps in underserved communities.

The limitations of the study are that it relies on formal datasets and this method may not adequately describe informal transport activities thus having a biasing effect on the assessment of transport access in the peripheral locations. This constraint is especially applicable to the African situation where informal transit systems can take a large proportion of daily journeys (54). Future studies should thus incorporate information regarding informal bus and paratransit services that play an essential role in such cities as Windhoek since both the formal and informal systems are present there. Based on this, health outcome measures should also be included in the future work. Linking TOD performance with localized health data, such as rates of respiratory diseases, obesity, or access to primary care, would powerfully quantify the public health impacts of planning interventions.

Furthermore, this study lays the groundwork for applying machine learning like the Random Forest model. Such approaches could quantify the influence of specific node-place indicators, identify critical TOD development thresholds, and uncover potential non-linear relationships between built environment factors and transit-oriented outcomes. These methodological advancements, combined with a more inclusive transport data framework, the integration of health metrics, and sophisticated analytical methods, will significantly enhance the precision and practical applicability of TOD assessments in developing cities.

## Data Availability

The raw data supporting the conclusions of this article will be made available by the authors, without undue reservation.

## References

[1] Capps KA Bentsen CN Ramírez A. Poverty, urbanization, and environmental degradation: urban streams in the developing world. Freshw Sci. (2016) 35:429–35. doi: 10.1086/684945, 12291371

[2] Nations U. World urbanization prospects: highlights. NYC, USA: United Nations (2018).

[3] Ruan Z Feng X Wu F Ding C Hua W. Land use and transport integration modeling with immune genetic optimization for urban transit-oriented development. J Urban Plann Dev. (2021) 147:04020063. doi: 10.1061/(ASCE)UP.1943-5444.0000658

[4] Appleyard BS Frost AR Allen C. Are all transit stations equal and equitable? Calculating sustainability, livability, health, and equity performance of smart growth and transit-oriented-development (TOD). J Transp Health. (2019) 14:100584. doi: 10.1016/j.jth.2019.100584, 41325008

[5] Almulhim AI Sharifi A Aina YA Ahmad S Mora L Filho WL . Charting sustainable urban development through a systematic review of SDG11 research. Nat Cities. (2024) 1:677–85. doi: 10.1038/s44284-024-00117-6

[6] Abubakar IR Aina YA. The prospects and challenges of developing more inclusive, safe, resilient and sustainable cities in Nigeria. Land Use Policy. (2019) 87:104105. doi: 10.1016/j.landusepol.2019.104105

[7] Brandt NL. Urban resilience and sustainability challenges: A case study on the city of Windhoek. Stellenbosch: South Africa Stellenbosch University (2019).

[8] Kohima JM Chigbu UE Mazambani ML Mabakeng MR. (neo-)segregation, (neo-)racism, and one-city two-system planning in Windhoek, Namibia: what can a new national urban policy do? Land Use Policy. (2023) 125:106480. doi: 10.1016/j.landusepol.2022.106480

[9] Robinson B Fisher R Windhoek, Namibia: Moving from transport planning to action. Washington DC: World Bank (2019)

[10] Bertolini L. Spatial development patterns and public transport: the application of an analytical model in the Netherlands. Plann Pract Res. (1999) 14:199–210. doi: 10.1080/02697459915724

[11] Liao C Scheuer B. Evaluating the performance of transit-oriented development in Beijing metro station areas: integrating morphology and demand into the node-place model. J Transp Geogr. (2022) 100:103333. doi: 10.1016/j.jtrangeo.2022.103333

[12] Gui W Zhang X Wang A. Research on spatial planning evaluation of Beijing Shanghai high speed railway station based on node-place model. J Intell Fuzzy Syst. (2021) 40:733–43. doi: 10.3233/JIFS-200712, 39743787

[13] Ali MM Alssadah GM Yu Y Altahir MM Damos MA Zhou R . Evaluating transit-oriented development (TOD) in Khartoum: a spatial analysis of bus terminals. J Urban Manag. (2025) 14:717–34. doi: 10.1016/j.jum.2025.01.010

[14] Li CX Yoon CJ. Analysis of urban rail public transport space congestion using graph fourier transform theory: a focus on Seoul. Sustainability. (2025) 17:598. doi: 10.3390/su17020598

[15] Sepe A. City planning with land value capture: a comparative analysis of capture policies in New York, London and Toronto. Canada: York University (2019)

[16] Liu Y Nitanai R Manabe R Murayama A. Institutionalization of transit-oriented development in Tokyo 1868-1945. Planning Perspect. (2023) 38:1185–212. doi: 10.1080/02665433.2023.2177184

[17] Guajardo Ortega MF Link H. Mode choice inertia and shock: three months of almost fare-free public transport in Germany. Econ Transp. (2025) 41:100382. doi: 10.1016/j.ecotra.2024.100382, 41325008

[18] Bartshe M Coughenour C Pharr J. Perceived walkability, social capital, and self-reported physical activity in Las Vegas college students. Sustainability. (2018) 10:3023. doi: 10.3390/su10093023

[19] Mackenbach JD Randal E Zhao P Howden-Chapman P. The influence of urban land-use and public transport facilities on active commuting in Wellington, New Zealand: active transport forecasting using the WILUTE model. Sustainability. (2016) 8:242. doi: 10.3390/su8030242

[20] Yang H Cui N Xia H. The evolution of the interaction between urban rail transit and land use: a citespace-based knowledge mapping approach. Land. (2025) 14:1386. doi: 10.3390/land14071386

[21] Calthorpe P. The next American Metropolis: Ecology, community, and the American dream, vol. 60. Abingdon, New York, NY: Princeton Architectural Press (1993) doi: 10.1016/j.tra.2019.10.018.

[22] Downs A. Smart growth: why we discuss it more than we do it. J Am Plan Assoc. (2005) 71:367–78. doi: 10.1080/01944360508976707

[23] Cervero R Kockelman K. Travel demand and the 3Ds: density, diversity, and design. Transp Res D Transp Environ. (1997) 2:199–219. doi: 10.1016/S1361-9209(97)00009-6

[24] Ewing R Cervero R. Travel and the built environment: a meta-analysis. J Am Plan Assoc. (2010) 76:265–94. doi: 10.1080/01944361003766766

[25] Wang Z Song J Zhang Y Li S Jia J Song C. Spatial heterogeneity analysis for influencing factors of outbound ridership of subway stations considering the optimal scale range of “7D” built environments. Sustainability. (2022) 14:16314. doi: 10.3390/su142316314

[26] Mangu S Kadali BR Subbarao SSV Lin J-J. Evaluation of transit-oriented development based on 9D’s approach in developing countries context. Transp Policy. (2025) 163:138–51. doi: 10.1016/j.tranpol.2025.01.010

[27] Xu W Guthrie A Fan Y Li Y. Transit-oriented development in China: literature review and evaluation of TOD potential across 50 Chinese cities. J Transp Land Use. (2017) 10:743–62. doi: 10.5198/jtlu.2017.922

[28] Xia J Zhang Y. Where are potential areas for transit-oriented development (TOD)-exploring the demands for built environment for TOD planning. Sustainability. (2022) 14:8364. doi: 10.3390/su14148364

[29] Geurs KT van Wee B. Accessibility evaluation of land-use and transport strategies: review and research directions. J Transp Geogr. (2004) 12:127–40. doi: 10.1016/j.jtrangeo.2003.10.005

[30] Masoumi Z van Genderen J. Artificial intelligence for sustainable development of smart cities and urban land-use management. Geo-spat Inf Sci. (2024) 27:1–25. doi: 10.1080/10095020.2023.2184729

[31] Ibraeva A de Almeida Correia GH Silva C Antunes AP. Transit-oriented development: a review of research achievements and challenges. Trans Res Pat A Policy Pract. (2019) 132:120–30. doi: 10.1016/j.tra.2019.10.018

[32] Huang J. A comparative study of transit-oriented development projects in China and the United States. Seattle, USA: University of Washington (2021).

[33] Li J Huang H. Effects of transit-oriented development (TOD) on housing prices: a case study in Wuhan, China. Res Transp Econ. (2020) 80:100813. doi: 10.1016/j.retrec.2020.100813

[34] Bertolini L. Nodes and places: complexities of railway station redevelopment. Eur Plan Stud. (1996) 4:331–45. doi: 10.1080/09654319608720349

[35] Yang Y Zhong C Gao QL. An extended node-place model for comparative studies of transit-oriented development. Transp Res D Transp Environ. (2022) 113:103514. doi: 10.1016/j.trd.2022.103514

[36] Cao Z Asakura Y Tan Z. Coordination between node, place, and ridership: comparing three transit operators in Tokyo. Transp Res D Transp Environ. (2020) 87:102518. doi: 10.1016/j.trd.2020.102518

[37] Zhao Y Hu S Zhang M. Evaluating equitable transit-oriented development (TOD) via the node-place-people model. Transp Res A Policy Pract. (2024) 185:104116. doi: 10.1016/j.tra.2024.104116

[38] Vale DS. Transit-oriented development, integration of land use and transport, and pedestrian accessibility: combining node-place model with pedestrian shed ratio to evaluate and classify station areas in Lisbon. J Transp Geogr. (2015) 45:70–80. doi: 10.1016/j.jtrangeo.2015.04.009

[39] Zhang M Lee JB. Make TOD more bicycling-friendly: an extended node-place model incorporating a cycling accessibility index. Buildings. (2023) 13:1240. doi: 10.3390/buildings13051240

[40] Zou Z Tang Y. Evaluation of sustainable development potential of high-speed railway station areas based on “node-place-industry” model. ISPRS Int J Geo Inf. (2023) 12:349. doi: 10.3390/ijgi12090349

[41] Zhang Y Marshall S Manley E. Network criticality and the node-place-design model: classifying metro station areas in greater London. J Transp Geogr. (2019) 79:102485. doi: 10.1016/j.jtrangeo.2019.102485

[42] Cervero R Dai D. BRT tod: leveraging transit oriented development with bus rapid transit investments. Transp Policy. (2014) 36:127–38. doi: 10.1016/j.tranpol.2014.08.005

[43] Zhao F Chow LF Li MT Ubaka I Gan AA. Forecasting transit walk accessibility: regression model alternative to buffer method. Transp Res Rec. (2003) 1835:34–41. doi: 10.3141/1835-05

[44] Wang L Xia H. A comprehensive review of the development characteristics and future trends of tod in Chinese urban rail transit. Urban Rail Transit. (2024) 10:335–49. doi: 10.1007/s40864-024-00228-2

[45] Sun B Ermagun A Dan B. Built environmental impacts on commuting mode choice and distance: evidence from Shanghai. Transp Res Part D Transp Environ. (2017) 52:441–53. doi: 10.1016/j.trd.2016.06.001

[46] Knight RL Trygg LL. Evidence of land use impacts of rapid transit systems. Transportation. (1977) 6:231–47. doi: 10.1007/BF00177453

[47] Salon D Sclar E Barone R. Can location value capture pay for transit? Organizational challenges of transforming theory into practice. Urban Aff Rev. (2019) 55:743–71. doi: 10.1177/107808741771552

[48] Shareef S Altan H. Sustainability at an urban level: a case study of a neighborhood in Dubai, UAE. Sustainability. (2021) 13:4355. doi: 10.3390/su13084355

[49] Almatar KM. Transit-oriented development in Saudi Arabia: Riyadh as a case study. Sustainability. (2022) 14:16129. doi: 10.3390/su142316129

[50] Dirgahayani P Syabri I Waluyo NP Land governance for transit oriented development in densely built urban area (case study: Jakarta, Indonesia). Proceedings of the 10th EASTS Conference (2015)

[51] Samad D Zainon N Rahim FAM Lou E. Malaysian affordability housing policies revisited. Open House Int. (2017) 42:44–51. doi: 10.1108/OHI-01-2017-B0007

[52] Tirumala RD Tiwari P. Importance of land in SDG policy instruments: a study of ASEAN developing countries. Land. (2022) 11:218. doi: 10.3390/land11020218

[53] van Ritsema Eck J Koomen E. Characterising urban concentration and land-use diversity in simulations of future land use. Ann Reg Sci. (2008) 42:123–40. doi: 10.1007/s00168-007-0141-7

[54] Asimeng ET Jauregui-Fung F. Tailoring bus rapid transit to the complex realities of African cities: critical issues and public policy planning approaches. Trans Res Interdisc Perspect. (2025) 30:101351. doi: 10.1016/j.trip.2025.101351

[55] Huang R Grigolon A Madureira M. Measuring transit-oriented development (TOD) network complementarity based on TOD node typology. J Transp Land Use. (2018). doi: 10.5198/jtlu.2018.1110

[56] Xia Z Zhang Y. From “5D” to “5D+N”: research published in English on the factors influencing TOD performance. Urban Plan Int. (2019) 34:109–16. doi: 10.22217/upi.2018.256

[57] Zhang M. Chinese edition of transit-oriented development. Transp Res Rec. (2007) 2038:120–7. doi: 10.3141/2038-16

[58] Ogra A Ndebele R The role of 6Ds: Density, diversity, design, destination, distance, and demand management in transit oriented development (TOD). Proceedings of the NICHE (2014)

